# Validation of the Patient Health Questionnaire-9 (PHQ-9) and PHQ-2 in patients with migraine

**DOI:** 10.1186/s10194-015-0552-2

**Published:** 2015-07-15

**Authors:** Jong-Geun Seo, Sung-Pa Park

**Affiliations:** Department of Neurology, School of Medicine, Kyungpook National University, 680 Gukchaebosang-ro, Jung-gu, Daegu, 700-842 Republic of Korea

**Keywords:** Depression, Migraine, PHQ-9, PHQ-2, Validity

## Abstract

**Background:**

Psychiatric problems have been commonly reported in patients with migraine. This study investigated the reliability and validity of the Patient Health Questionnaire-9 (PHQ-9) and Patient Health Questionnaire-9 (PHQ-2) in patients with migraine.

**Methods:**

Patients with migraine (with or without aura) were consecutively recruited from our headache clinic. They completed several instruments, including the Mini International Neuropsychiatric Interview-Plus Version 5.0.0 (MINI), the PHQ-9, the Beck Depression Inventory-II (BDI-II), the Migraine Disability Assessment Scale (MIDAS), the Headache Impact Test-6 (HIT-6), and the Migraine-Specific Quality of Life (MSQoL).

**Results:**

Among 132 participants, 39 patients (29.5 %) had a major depressive disorder (MDD) as determined by the MINI. Cronbach’s *α* coefficients for the PHQ-9 and PHQ-2 were 0.894 and 0.747, respectively. At a cutoff score of 7, the PHQ-9 had a sensitivity of 79.5 %, a specificity of 81.7 %, a positive predictive value (PPV) of 64.6 %, and a negative predictive value (NPV) of 90.5 %. At a cutoff score of 2, the PHQ-2 had a sensitivity of 66.7 %, a specificity of 90.3 %, a PPV of 74.3 %, and a NPV of 86.6 %. The scores of the PHQ-9 and PHQ-2 well correlated with the BDI-II score, the MIDAS score, the HIT-6 score, and the MSQoL score.

**Conclusions:**

The PHQ-9 and PHQ-2 are both reliable and valid screening instruments for MDD in patients with migraine.

## Background

Approximately 10–15 % of the general population is affected by migraines, which are characterized by recurrent attacks of severe pulsating headaches lasting 4–72 h [1]. Migraine is the sixth highest cause of disability worldwide [2]. Patients with migraine are more likely to develop depression than those without migraine. In a review of the literature, the prevalence of depression varied from 8.6 to 47.9 % in patients with migraine [3]. The overall risk of developing depression was 2.2 times higher in patients with migraine [3].

Comorbidity with psychiatric disorders raises the global burden of migraine. Disability and health related quality of life (HRQoL) impairment in patients with migraine is greater when migraine is associated with either depression or anxiety [4, 5]. Individuals with migraine and comorbid psychiatric disorders use more health resource than those with migraine alone [6]. In addition, the presence of psychiatric problems is a risk factor for transformation to chronic form of migraine [6] and seem to play a role in the evolution of migraine to medication overuse headache (MOH) [7]. Therefore, the early diagnosis and treatment of depression is important for the proper management of patients with migraine. For these purposes, a simple, rapid screening instrument to detect depression is a prerequisite, especially in a busy clinical setting.

The Patient Health Questionnaire-9 (PHQ-9) is a valuable screening instrument for detecting a major depressive disorder (MDD) [8] based on diagnostic criteria from the Diagnostic and Statistical Manual of Mental Disorders, Fourth Edition (DSM-IV) [9], and it is generally useful in headache studies [10]. The Patient Health Questionnaire-2 (PHQ-2), which includes the first two items of the PHQ-9, is also a valuable instrument [11]. Although these instruments were validated in primary care patients, their usefulness in patients with migraine is unknown. Therefore, the aim of this study was to investigate the value of the PHQ-9 and PHQ-2 as screening tools in patients with migraine.

## Methods

### Subjects

Subjects in this study were patients with consecutive visits to the headache clinic in the Department of Neurology at Kyungpook National University Hospital between April and November of 2014. The patients, ranging from 16 to 70 years of age, all had a current diagnosis of migraine and did not take preventive medicines for migraine or other psychotropic agents. A diagnosis of migraine was based on the International Classification of Headache Disorders, 3^rd^ edition, beta version by a trained neurologist (S.P. Park) [12]. Patients were excluded if they were unable to cooperate in the psychiatric interview or had difficulty understanding the questionnaire because of illiteracy, mental retardation, serious medical, neurological, or psychiatric disorders, and alcohol or drug abuse. Patients with a probable migraine and those declining the interview were also excluded.

### Study design

A cross-sectional study was approved by the institutional review board of Kyungpook National University Hospital, and all subjects provided written informed consent prior to the study. Patients were interviewed by S.P. Park, who also reviewed the medical charts to collect demographic, social, and clinical information for a database. Sociodemographic data included age, gender, education, employment, household income (earning more or less than three million KRW per month, equivalent to 2,800 USD per month), and marital states (married, unmarried, divorced, and bereaved). Clinical data included the type of migraine, migraine chronicity (episodic migraine [EM] or chronic migraine [CM]), MOH, age at onset, disease duration, attack frequency, attack duration, and family history. A family history of migraine was defined as an existing diagnosis of migraine in a lineal ascendant and siblings.

To measure the reliability of the PHQ-9 and PHQ-2 in eligible subjects, a neuropsychologist examined their MDD using the Mini International Neuropsychiatric Interview-Plus Version 5.0.0 (MINI) [13]. Subsequently, patients provided several self-reported questionnaires, including the Beck Depression Inventory-II (BDI-II) [14], the Migraine Disability Assessment Scale (MIDAS) [15], the Headache Impact Test-6 (HIT-6) [16], and the Migraine-Specific Quality of Life (MSQoL) [17], to examine the validity of the PHQ-9 and PHQ-2.

### Interview and questionnaires

#### Mini International Neuropsychiatric Interview-Plus Version 5.0.0 (MINI)

The MINI is an internationally validated brief structured interview used extensively as a diagnostic tool for psychiatric disorders from the DSM-IV and the International Classification of Diseases-10. The reliability and validity of this instrument is well established [18], and the Korean translation is also validated [13]. The Kappa value of MDD was 0.71, indicating a moderate and substantial agreement between the MINI and the expert’s diagnoses.

#### Patient Health Questionnaire-9 (PHQ-9) and Patient Health Questionnaire-2 (PHQ-2)

The PHQ-9 and PHQ-2 were designed for use in primary care patients [8, 11]. The PHQ-9 includes nine items pertaining to the DSM-IV criteria for MDD [9]: (1) anhedonia; (2) depressed mood; (3) trouble sleeping; (4) feeling tired; (5) change in appetite; (6) guilt, self-blame, or worthlessness; (7) trouble concentrating; (8) feeling slowed down or restless; and (9) thoughts of being better off dead or hurting oneself [8]. Each item is rated on a 4-point scale from 0 to 3 (0 - never; 1 – several days; 2 - more than half the time; and 3 - nearly every day) during the two weeks prior to and including the day of survey completion. The overall scores ranged from 0 to 27. At a cutoff score of 9, the PHQ-9 had a sensitivity of 88 % and a specificity of 88 % for detecting MDD compared with a structured psychiatric interview [8]. The PHQ-2 includes only the first two items in the PHQ-9, which are critical for the diagnosis of MDD [11]. The overall scores ranged from 0 to 6. At a cutoff score of 2, the PHQ-2 had a sensitivity of 83 % and a specificity of 92 % for detecting MDD [11]. The PHQ-9 was translated into Korean language, and was freely downloadable on the PHQ website (http://www.phqscreeners.com/) [19]. The translated version was back translated into English by a Korean English teacher. Finally, the two versions were compared by a native English speaker who concluded that they were identical. Thereafter, we administered it to 20 Korean patients with migraine to evaluate potential problems in comprehension or cultural differences. No further adaptations were required.

#### Beck Depression Inventory-II (BDI-II)

The BDI-II is a commonly used self-rating scale for depression symptoms [20]. Patients score 21 items on a scale from 0 to 3 according to how they felt during the previous 2 weeks. The total scores ranged from 0 to 63. The Korean version of the BDI-II has been validated [14]. Cronbach’s α coefficient was 0.834 in depressive patients and 0.88 in healthy subjects. At a cutoff score of 22, the BDI-II had a sensitivity of 94 % and specificity of 98 % for detecting MDD compared with a structured psychiatric interview.

#### Migraine Disability Assessment Scale (MIDAS)

The Korean version of the MIDAS, a five-item questionnaire designed to evaluate disability within during the previous 3 months, was used in this study [15]. Patients were asked to report decreased performance in the domains of work/school, household work, and family/social activities. Scores (0–27) measure the overall level of disability: Grade I (0–5), Grade II (6–10), Grade III (11–20), and Grade IV (above 21). Cronbach’s α value was 0.75.

#### Headache Impact Test-6 (HIT-6)

The HIT-6 was developed in the United States to measure a wider spectrum of headache-induced burden [21]. Items in the HIT-6 cover several HRQOL domains: pain, social functioning, role functioning, vitality, cognitive functioning, and psychological distress. Each item is answered on a 5-point Likert scale (6 = never, 8 = rarely, 10 = sometimes, 11 = very often, 13 = always). The total scores ranged from 36 to 78; larger scores indicate greater impact. For interpretation, HIT-6 scores are categorized in four groups: scores of ≤49 indicate little or no impact; scores between 50 and 55 indicate some impact; scores between 56 and 59 indicate a substantial impact; and scores ≥60 indicate a severe impact [22]. The Korean version of the HIT-6 was validated and Cronbach’s α coefficient was 0.85 [16].

#### Migraine-Specific Quality of Life (MSQoL)

The MSQoL, developed by Wagner et al., is a valid and reliable tool for clinical migraine research [23]. A Korean translation of this 25-item questionnaire has been validated [17]. The items are rated on a 4-point scale (1–4). The total scores ranged from 25 to 100. A lower total score indicates poorer QOL. Cronbach’s α value was 0.93.

### Statistical analyses

The Statistical Package for the Social Sciences (SPSS version 21.0) was used for data analysis. The Med Calc 8.0 was used to perform receiver operating characteristic (ROC) analyses, which measure sensitivity, specificity, positive predictive values (PPVs) and negative predictive values (NPVs). ROC analyses for the PHQ-9 and PHQ-2, over a range of cutoff scores, were performed for comparison to MDD diagnoses by the MINI. Optimal cutoff scores were also computed using criteria that minimize the Euclidean distance from point (sensitivity and specificity) to point in the x-y plane. Descriptive statistics are presented as counts, percentages, means, and standard deviations. Independent t tests, Mann–Whitney U tests, and Chi-square tests were used to compare continuous or categorical variables. Cronbach’s α coefficient was computed to ascertain internal consistency and was recalculated after items were removed. Nonparametric correlations (Spearman’s *ρ*) were used to determine the validity of the PHQ-9 and PHQ-2. The level of statistical significance was set at *p* < 0.05.

## Results

Of the 185 patients who consecutively visited our headache clinic, 53 were excluded due to probable migraine (n = 21), taking preventive medicines for migraine or psychotropic agents (n = 10), illiteracy (*n* = 5), age older than 70 (*n* = 3), and refusal to take part in the study (*n* = 14). The 132 remaining patients were eligible for this study. Of them, 73 patients (55.3 %) had CM and 36 patients (27.3 %) exhibited MOH. According to the MINI, 39 patients (29.5 %) were diagnosed with MDD. The relationship between MDD demographic, clinical, and psychosocial characteristics are listed in Table 1. Patients with MDD were less likely to be employed and more likely to have a low household income than those without MDD. Patients with MDD had a higher risk of developing CM and phonophobia than those without MDD. Patients with MDD exhibited higher scores on the PHQ-9, the BDI-II, the MIDAS, and the HIT-6, a lower score on the MSQoL than those without MDD.

**Table 1:** Demographic, clinical, and psychosocial characteristics of the eligible subjects with respect to current MDD as determined by the MINI-Plus 5.0.0

	Mean ± SD (range) or number (%)		
	No MDD	MDD	
Characteristics	(*n* = 93)	(*n* = 39)	*p* value*
Age, years	40.3 ± 12.6 (16–65)	38.6 ± 14.0 (17–70)	0.495
Gender, female	81 (87.1)	33 (84.6)	0.705
Education, years	13.3 ± 2.7 (6–18)	12.6 ± 2.9 (6–18)	0.204
Job, yes	49 (52.7)	26 (66.7)	0.042
Household income, at least 3 million KRW/month	69 (74.2)	20 (51.3)	0.010
Married without divorce or bereavement	64 (68.8)	20 (51.3)	0.056
**Type of migraine**			**0.305**
**Migraine with aura**	**10 (10.8)**	**2 (5.1)**	
**Migraine without aura**	**83 (89.2)**	**37 (94.9)**	
**Migraine chronicity**			**0.007**
**Episodic migraine**	**49 (52.7)**	**10 (25.6)**	
**Chronic migraine**	**44 (47.3)**	**29 (74.4)**	
**MOH**	**23 (24.7)**	**13 (33.3)**	**0.392**
Age at onset, years	30.3 ± 11.2 (8–52)	30.1 ± 12.2 (9–53)	0.947
Disease duration, years	10.0 ± 7.8 (0–37)	8.5 ± 7.8 (0–33)	0.293
Attack frequency/3 months	17.1 ± 21.5 (1–90)	23.7 ± 21.6 (1–70)	0.113
Attack duration, hours	31.0 ± 23.6 (4–72)	29.4 ± 22.1 (4–72)	0.717
Family history of migraine	65 (69.9)	24 (61.5)	0.350
Photophobia	42 (45.2)	22 (56.4)	0.238
Phonophobia	58 (62.4)	32 (82.1)	0.027
Osmophobia	41 (44.1)	19 (48.7)	0.626
PHQ-9 score	4.5 ± 3.4 (0–15)	13.4 ± 6.8 (1–27)	<0.001
BDI-II score	12.0 ± 7.3 (0–34)	30.4 ± 12.4 (9–56)	<0.001
MIDAS, day	16.3 ± 20.7 (0–100)	37.7 ± 40.5 (0–183)	0.003
HIT-6 score	57.8 ± 8.4 (36–78)	63.8 ± 7.3 (48–78)	<0.001
MSQoL	69.0 ± 15.8 (34–99)	57.1 ± 16.2 (26–90)	<0.001

*MDD* Major Depressive Disorder, *MINI-Plus 5.0.0* Mini International Neuropsychiatric Interview-Plus Version 5.0.0, *KRW* Korean Won, *MOH* medication overuse headache, *PHQ-9* Patient Health Questionnaire-9, *BDI-II* Beck Depression Inventory-II, *MIDAS* Migraine Disability Assessment Scale, *HIT-6* Headache Impact Test-6, *MSQoL* Migraine-Specific Quality of Life

^*^Independent *t-*test or chi-square tests were performed for the comparison of variables

**Fig. 1:**
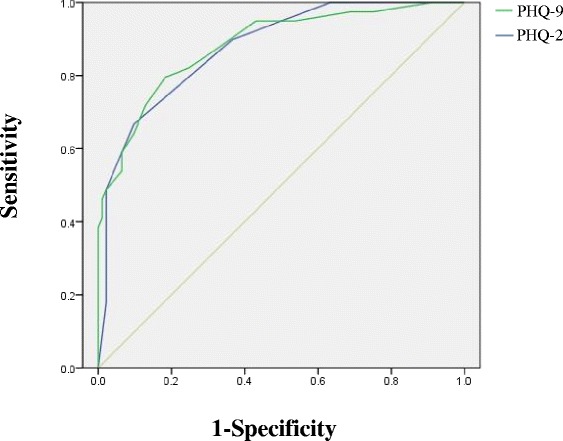
Receiver operating characteristic curves of the Patient Health Questionnaire-9 and Patient Health Questionnaire-2

The subjects completed the PHQ-9 without any difficulties in comprehending and replying to the questions. Cronbach’s α coefficients for the PHQ-9 and PHQ-2 were 0.894 and 0.747, respectively, indicating excellent internal consistency. As shown in Table 2, all items in the PHQ-9 were significantly and positively associated with the total PHQ-9 score, and the α did not decrease if items were deleted. The ROC analyses of the PHQ-9 and PHQ-2 are shown in Table 3 and the ROC curves are illustrated in Fig. 1. ROC analysis of the PHQ-9 determined an area under the curve (AUC) of 0.882 (95 % CI = 0.818-0.947; SE = 0.033; *p* < 0.001). At a cutoff score of >7, the PHQ-9 sensitivity was 79.5 % and specificity was 81.7 %, with a PPV of 64.6 % and an NPV of 90.5 %. For our patients, the MDD frequency was 36.4 % using a cutoff score of 7. ROC analysis of the PHQ-2 revealed an AUC of 0.876 (95 % CI = 0.814–0.938; SE = 0.032; *p* < 0.001). At a cutoff score >2, the PHQ-2 sensitivity was 66.7 % with a specificity of 90.3 %, a PPV of 74.3 %, and a NPV of 86.6 %. MDD frequency was 26.5 % at a cutoff score of 2.

**Table 2:** Corrected item-total correlations and Cronbach’s *α* when an item is deleted from the PHQ-9

	Corrected item-total correlation	Cronbach’s α if an item deleted
Item 1	0.734	0.876
Item 2	0.667	0.881
Item 3	0.563	0.892
Item 4	0.727	0.876
Item 5	0.713	0.878
Item 6	0.716	0.878
Item 7	0.607	0.886
Item 8	0.607	0.887
Item 9	0.641	0.885

*PHQ-9* Patient Health Questionnaire-9

**Table 3:** ROC analyses of the PHQ-9 and PHQ-2 for the diagnosis of current MDD as determined by the MINI-Plus 5.0.0

Cut off score	Sensitivity	Specificity	PPV	NPV	AUC	SE	95 % CI	*p* value
PHQ-9								
>5	87.2	67.3	53.1	92.6	0.775	0.043	0.690–0.859	<0.001
>6	82.1	75.3	58.2	90.9	0.787	0.044	0.700–0.873	<0.001
>7	79.5	81.7	64.6	90.5	0.806	0.044	0.720–0.892	<0.001
>8	71.8	87.1	70.0	88.0	0.794	0.047	0.702–0.887	<0.001
>9	64.1	90.3	73.5	85.7	0.772	0.050	0.674–0.870	<0.001
PHQ-2								
>1	89.7	63.4	50.7	93.7	0.766	0.043	0.682–0.850	<0.001
>2	66.7	90.3	74.3	86.6	0.785	0.049	0.689–0.881	<0.001
>3	48.7	97.8	90.5	82.0	0.733	0.054	0.626–0.839	<0.001

*ROC* Receiver operating characteristic, *PHQ-9* Patient Health Questionnaire-9, *PHQ-2* Patient Health Questionnaire-2, *MDD* Major Depressive Disorder, *MINI-Plus 5.0.0* Mini International Neuropsychiatric Interview-Plus Version 5.0.0, *PPV* positive predictive value, *NPV* negative predictive value, *AUC* area under the curve

The validity of the PHQ-9 and PHQ-2 are shown in Table 4. The PHQ-9 score is well correlated with the BDI-II score (Spearman’s *ρ* = 0.754, *p* < 0.001), the MIDAS score (Spearman’s *ρ* = 0.377, *p* < 0.001), the HIT-6 score (Spearman’s *ρ* = 0.519, *p* < 0.001), and the MSQoL score (Spearman’s *ρ* = −0.538, *p* < 0.001). The PHQ-2 score was also well correlated with the BDI-II score (Spearman’s *ρ* = 0.739, *p* < 0.001), the MIDAS score (Spearman’s *ρ* = 0.396, *p* < 0.001), the HIT-6 score (Spearman’s *ρ* = 0.556, *p* < 0.001), and the MSQoL score (Spearman’s *ρ* = −0.580, *p* < 0.001).

**Table 4:** Correlation between the PHQ-9 and PHQ-2 scores and the BDI-II score, the MIDAS score, the HIT-6 score, and the MSQoL score

Variable	*r*	*p* value^a^
PHQ-9		
BDI-II	0.754	<0.001
MIDAS	0.377	<0.001
HIT-6	0.519	<0.001
MSQoL	−0.538	<0.001
PHQ-2		
BDI-II	0.739	<0.001
MIDAS	0.396	<0.001
HIT-6	0.556	<0.001
MSQoL	−0.580	<0.001

*PHQ-9* Patient Health Questionnaire-9, *PHQ-2* Patient Health Questionnaire-2, *BDI-II* Beck Depression Inventory-II, *MIDAS* Migraine Disability Assessment Score, *HIT-6* Headache Impact Test-6, *MSQoL* Migraine-Specific Quality of Life

^a^Spearman correlations are applied

## Discussion

To our knowledge, this is the first study investigating the usefulness of the PHQ-9 and PHQ-2 as screening instruments in patients with migraine. We found that the PHQ-9 and PHQ-2 were easily comprehended and quickly completed by the patients. Furthermore, they had excellent internal consistency reliability (Cronbach’s *α* =0.894 for the PHQ-9 and Cronbach’s *α* =0.747 for the PHQ-2). The validity of the PHQ-9 and PHQ-2 was determined by correlation with scores from the BDI-II, the MIDAS, the HIT-6, and the MSQoL. Together, these data suggest that both the PHQ-9 and PHQ-2 are useful screening instruments for the diagnosis of MDD in patients with migraine.

Although there has not yet been a study to validate the PHQ-9 in patients with migraine, many validation studies have been conducted for patients in primary care and hospital settings. The initial validation study for the PHQ-9, conducted in primary care patients, had a Cronbach’s *α* of 0.89, a sensitivity of 88 %, and a specificity of 88 % at a cutoff score of 9 [8]. In a Korean study of primary care patients, Cronbach’s *α* was 0.852, sensitivity was 90.9 % and specificity was 87 % using a cutoff score of 8 [24]. While the reliability in our study is consistent with these reports, the sensitivity, specificity, and cutoff scores were all lower. A 2012 meta-analyses included eighteen validation studies from primary care, specialized secondary care services (brain injury, cardiology, stroke, and nephrology), and the community [25]. Eleven of the studies provided details about the diagnostic properties of the questionnaire and the pooled sensitivity ranged from 62 % with a cutoff score of 14 to 89 % using a cutoff score of 10. Pooled specificity results ranged from 73 % with a cutoff score of 6 to 96 % with a cutoff score of 14 [25]. There were no substantial differences in the pooled sensitivity and specificity for cutoff scores from 7 to 10. The cutoff score, sensitivity, and specificity of the PHQ-9 determined in our study are consistent with the literature.

The PHQ-2 has not been as frequently validated as the PHQ-9. The initial PHQ-2 validation study was conducted on primary care patients and it reported a sensitivity of 83 % and specificity of 92 % at a cutoff score of 2 [11]. A Korean study in a tertiary care hospital determined a sensitivity of 91.9 % and specificity of 100 % at a cutoff score of 2 [26]. In a neurologic field, a validation study for patients with Parkinson’s disease documented a sensitivity of 75 % and a specificity of 89 % at a cutoff score of 2 [27]. In comparison, our study established a lower sensitivity for the PHQ-2 at the same cutoff score. Our study also showed that the PHQ-2 had a lower sensitivity and a higher specificity than the PHQ-9. Therefore, we should be cautious interpreting results of the PHQ-2 when establishing the frequency of MDD in patients with migraine.

We reported that MDD frequency was 36.4 % when we applied a cutoff score of 7. However, the frequency was 25.8 % when we used a cutoff score of 9 in the initial validation study [8]. Using a cutoff score of 9 excludes 10.6 % of the patients from the diagnosis of MDD. This suggests that the PHQ-9 validation should be performed for each study settings (primary care setting or hospital setting) and specific disease groups. For example, a validation study of the PHQ-9 for patients with Parkinson’s disease in the US reported that a cutoff score of 5 was appropriate for detecting MDD [28]. If a cutoff score of 9 were applied to these patients, many would be excluded from the diagnosis of MDD. We also recommend validating the PHQ-9 for use in different languages and countries due to the linguistic characteristics of each country. For example, a rapid screening instrument for detecting MDD in people with epilepsy, the Neurological Disorders Depression Inventory for Epilepsy, had different cutoff scores when it was validated in different languages [29]. Differences in the cutoff score may also be influenced by cross-cultural differences during validation. For example, individuals with an Asian background are more likely to express themselves conservatively, leading to the possibility that Korean patients with migraine are less likely to report depression symptoms. Given these possibilities, we should encourage clinicians to translate and validate the PHQ-9 according to specific diseases, native languages, and cultural differences.

There are several limitations to our study. First, the PHQ-9 and PHQ-2 provide only a probable diagnosis of MDD that should be investigated by further evaluation. Second, a cutoff score of 7 in the PHQ-9 had a PPV of 64.6 %, which may lead to false-positive results. Third, our study validated the Korean version of the PHQ-9 and PHQ-2 in Korean patients with migraine and their diagnostic properties may be different from those in other languages and countries.

## Conclusions

Patients with migraine are more likely to develop depression than those without migraine [3]. Comorbid depression in patients with migraine may have important clinical implications.

In a busy clinical setting, psychiatric interviews take a long time to conduct. Therefore, the application of the PHQ-9 and PHQ-2 could lead to a better recognition of depression in patients with migraine. Furthermore, because the PHQ-9 and PHQ-2 are quite simple and brief, they could be useful to detect the presence of depression in many neurologic disorders.

## Abbreviations

HRQoL: Health related quality of life
MOH: medication overuse headache
PHQ: Patient Health Questionnaire
MDD: Major depressive disorder
DSM-IV: Diagnostic and Statistical Manual of Mental Disorders, Fourth Edition
EM: episodic migraine
CM: Chronic migraine
MINI: Mini International Neuropsychiatric Interview
BDI: Beck Depression Inventory
MIDAS: Migraine Disability Assessment Scale
HIT: Headache Impact Test
MSQoL: Migraine-Specific Quality of Life
ROC: Receiver operating characteristic
PPVs: Positive predictive values
NPVs: Negative predictive values
